# Currently used dosage regimens of vancomycin fail to achieve therapeutic levels in approximately 40% of intensive care unit patients

**DOI:** 10.5935/0103-507X.20160071

**Published:** 2016

**Authors:** Vitor Yuzo Obara, Carolina Petrus Zacas, Claudia Maria Dantas de Maio Carrilho, Vinicius Daher Alvares Delfino

**Affiliations:** 1 Postgraduate Program in Health Sciences, Universidade Estadual de Londrina - Londrina (PR), Brazil.; 2 Universidade Estadual de Londrina - Londrina (PR), Brazil.

**Keywords:** Vancomycin/administration & dosage, Vancomycin/pharmacokinetics, Bacterial infections/therapy, Vancomicina/administração & dosagem, Infecções por bactérias Gram-positivas/quimioterapia, Lesão renal aguda/quimioterapia

## Abstract

**Objective:**

This study aimed to assess whether currently used dosages of vancomycin for treatment of serious gram-positive bacterial infections in intensive care unit patients provided initial therapeutic vancomycin trough levels and to examine possible factors associated with the presence of adequate initial vancomycin trough levels in these patients.

**Methods:**

A prospective descriptive study with convenience sampling was performed. Nursing note and medical record data were collected from September 2013 to July 2014 for patients who met inclusion criteria. Eighty-three patients were included. Initial vancomycin trough levels were obtained immediately before vancomycin fourth dose. Acute kidney injury was defined as an increase of at least 0.3mg/dL in serum creatinine within 48 hours.

**Results:**

Considering vancomycin trough levels recommended for serious gram-positive infection treatment (15 - 20µg/mL), patients were categorized as presenting with low, adequate, and high vancomycin trough levels (35 [42.2%], 18 [21.7%], and 30 [36.1%] patients, respectively). Acute kidney injury patients had significantly greater vancomycin trough levels (p = 0.0055, with significance for a trend, p = 0.0023).

**Conclusion:**

Surprisingly, more than 40% of the patients did not reach an effective initial vancomycin trough level. Studies on pharmacokinetic and dosage regimens of vancomycin in intensive care unit patients are necessary to circumvent this high proportion of failures to obtain adequate initial vancomycin trough levels. Vancomycin use without trough serum level monitoring in critically ill patients should be discouraged.

## Abstract (pt)

**Objetivo:**

Avaliar se a posologia atualmente utilizada de vancomicina para tratamento de infecções bacterianas graves causadas por microrganismos *Gram*-positivos em pacientes admitidos à unidade de terapia intensiva proporcionam níveis plasmáticos de vale de vancomicina em nível terapêutico, e examinar possíveis fatores associados com níveis de vale de vancomicina adequados nesses pacientes.

**Métodos:**

Estudo prospectivo descritivo com amostra de conveniência. Os pacientes que cumpriam os critérios de inclusão tiveram seus dados coletados a partir das anotações da enfermagem e dos registros médicos entre setembro de 2013 e julho de 2014. Incluíram-se 83 pacientes. Os níveis plasmáticos de vale iniciais de vancomicina foram obtidos imediatamente antes da quarta dose de vancomicina. Definiu-se lesão renal aguda como um aumento de, pelo menos, 0,3mg/dL na creatinina sérica dentro de 48 horas.

**Resultados:**

Considerando os níveis de vale plasmáticos de vancomicina recomendados para o tratamento de infecções graves por *Gram*-positivos (15 - 20µg/mL), os pacientes foram categorizados em grupos como níveis de vale de vancomicina baixos, adequados e elevados, respectivamente divididos em 35 (42,2%), 18 (21,7%), e 30 (36,1%) pacientes. Os pacientes com lesão renal aguda tiveram níveis plasmáticos de vale de vancomicina significantemente mais elevados (p = 0,0055, com significância para tendência, p = 0,0023).

**Conclusão:**

Preocupantemente, mais de 40% dos pacientes não obtiveram níveis plasmáticos de vale de vancomicina considerados eficazes. São necessários estudos de farmacocinética e de regimes posológicos de vancomicina em pacientes admitidos em unidades de terapia intensiva, para contornar esta elevada proporção de falhas na obtenção de níveis de vale iniciais adequados de vancomicina. Deve ser desencorajado o uso de vancomicina sem monitoramento dos níveis de vale plasmáticos.

## INTRODUCTION

Vancomycin (vanco) is glycopeptide antibiotic that has been used clinically for over 50 years. It remains recommended as a first-line agent for severe infections caused by methicillin-resistant *Staphylococcus aureus* (MRSA).^(1)^ In recent years, the minimum inhibitory concentration (MIC) of vanco has increased in some parts of the world,^(2,3)^ Brazil included.^(4)^

When using vanco in the treatment of serious infections caused by MRSA, it is recommend that the pharmacokinetic-pharmacodynamic target for area under the curve of serum concentration by 24 hours and the MIC ratio (AUC/MIC) should be greater than 400.^(5)^

Due to difficulties inherent in obtaining AUC/MIC (it is not feasible in clinical practice), the Infectious Diseases Society of America (ISDA), the American Society of Health-System Pharmacists, and the Infectious Diseases Society of Pharmacists suggest monitoring serum vanco trough levels (VTLs) to guide the antibacterial therapy, due to its practicality in presenting a good correlation with the AUC/MIC and with the recommendation that VTLs be maintained between 15 µg/mL to 20µg/mL, avoiding levels lower than 10µg/mL.^(5)^ The ISDA, as well other authors, have recommend that VTL should be measured within 30 minutes prior to infusion of the fourth or fifth dose of vanco following the initial dose or a dose adjustment.^(1,6)^ Authors of a recent systematic review and meta-analysis concluded that adherence to these recommended therapeutic levels for vanco was associated with better clinical results and lower nephrotoxicity.^(7)^

However, intensive care unit (ICU) patients may present with factors that either increase (as an augmented glomerular filtration rate [GFR]) or decrease (acute kidney injury [AKI] and hypoalbuminemia, which increases unbound drug concentration) vanco clearance.^(8)^ Moreover, the pharmacokinetics of vanco in these patients are often altered due to an increased volume of distribution, which can lead to reduced serum levels of this antibiotic.^(9)^

This study aimed to assess whether currently used dosages of vanco for the treatment of serious gram-positive bacterial infections in intensive care units patients of a southern Brazilian university hospital provided initial therapeutic vanco trough levels and to examine possible factors associated with the presence of adequate initial vanco trough levels in these patients.

## METHODS

This observational prospective descriptive study was conducted in two ICUs at the *Hospital Universitário de Londrina*, Brazil. Convenience sampling included all consecutive patients older than 18 years admitted from September 2013 to July 2014 at the *Hospital Universitário de Londrina* ICU who were treated with vanco and had at least one VTL available. Pregnant women, patients without a baseline serum creatinine level, patients with serum creatinine > 2.0mg/dL at vanco prescription, patients with chronic kidney diseases or those having been submitted to hemodialysis prior to the use of vanco were excluded. The study was approved by the *Universidade Estadual de Londrina*'s Ethics Committee, based on opinion 492785/2013, Presentation to Ethics Assessment Certificate (CAAE): 17118313.0.0000.5231. All patients (or their legal representative) participating in the study signed the informed consent form.

Vanco was empirically suggested for severely ill patients: a loading dose of 25 - 30mg/kg and a daily dose of 15 - 20mg/kg of estimated weight q6 h to q12 h, with a maximum single dose of 2g and a maximum daily dose of 6g.^(6,10)^ Adjustments in vanco dose intervals were made if the patient's GFR estimated by the 4 variable Modification of Diet in Renal Disease equation study were less than 50mL/min/1.73m^2^.^(11)^ Blood samples for the initial VTL were drawn immediately before the fourth dose of vanco. The patient's data at ICU admission were used to calculate the Acute Physiology and Chronic Health Disease Classification System II - APACHE II score.^(12)^ From the initial use of vanco until the first VTL dosage, clinical and laboratory notes were recorded. Sequential Organ Failure Assessment (SOFA)^(13)^ scores were calculated from these notes. VTL and serum creatinine dosages were determined by Dimension^®^ Clinical Chemistry System from Siemens. VTLs were categorized as low (< 15µg/mL), adequate (15 to 20µg/mL), or high (> 20µg/mL)^(14)^ After the results of initial and subsequent VTLs, modifications in vanco dose or administration intervals needed to maintain a VTL within the adequate levels were done in accordance with a vancomycin dosage adjustment monograph.^(6)^ Acute kidney injury was defined by an elevation of at least 0.3mg/dL in serum creatinine within 48 hours, followed by one of the Kidney Disease Improving Global Outcomes (KDIGO) criteria for AKI definition.^(15)^ KDIGO staging criteria based on serum creatinine and dialysis needed were applied as the following: stage I (increase of at least 0.3mg/dL within 48 hours); Stage II (increase of 2 to 2.9 times the baseline value); Stage III (increase of 3 times or more than the baseline value, serum creatinine ≥ 4mg/dL or higher, or early dialysis). Shock was indirectly assessed by the need for vasoactive drugs and respiratory failure was assessed by the need for mechanical ventilation.

Data were analyzed using descriptive statistics using the means and standard deviations or medians and interquartile ranges, as dictated by the normality of data. Differences between groups were analyzed by the Kruskal-Wallis method followed by Dunn's test, a Mann-Whitney test, and an unpaired *t* test, when applicable. Proportions of patients who developed AKI, sex, and vasoactive drug and mechanical ventilation use were analyzed with a chi-squared test for independence or a chi-squared test for trend. Stuart-Maxwell's test was also used to compare the proportion of patients with a low, adequate or high VTL over time. All statistical tests were performed using R Development Core Team (2011), and the Stuart-Maxwell's test was performed with 'coin' package and Dunn's test, with 'dunn.test'. All tests were two-tailed, and a p-value < 0.05 was considered to be statistically significant.

## RESULTS

During the study period, 135 ICU patients were included. Fifty-two were excluded due to the following reasons: age below 18 years (n = 3), early discharge from the ICU (n = 4), prior hemodialysis (n = 7), vanco suspended before first VTL determination (n = 10), baseline serum creatinine greater than 2.0 (n = 13), death (n = 10), and other causes (n = 5). Thus, 83 patients remained to be studied. Clinical and laboratory baseline characteristics of these 83 patients stratified according to VTL are presented in table 1.

**Table 1:** Basal clinical and laboratory characteristics of patients distributed into vancomycin trough level categories

	Low N = 35	Adequate N = 18	High N = 30	p value
Age	53.0 (29.0 - 64.0)	64.5 (52.3 - 79.5)	55.5 (44.0 - 70.8)	0.0657
Sex, male	24 (68.6)	12 (66.7)	18 (60.0)	0.7603
Weight (kg)	70 (67.5 - 80.0)	70 (65 - 70)	75 (70 - 80)	0.0395*
Serum creatinine (mg/dL)	0.90 (0.80 - 1.25)	1.05 (0.80 - 1.18)	1.00 (0.9 - 1.40)	0.3881
eGFR (mL/min/1.73 m^2^)	84.9 (73.3 - 96.4)	74.8 (59.4 - 90.1)	70.7 (60.2 - 81.2)	0.1794
AKI (KDIGO)	8(22.9)	5(27.8)	18(60.0)	0.0055 (0.0023 for trend)†
AKI stage	I: 6 (17.1)	I: 3 (16.7)	I: 8 (26.7)	-
	II: 1 (2.9)	II: 1 (5.6)	II: 4 (13.3)	
	III: 1 (2.9)	III: 1 (5.6)	III: 6 (20.0)	
APACHE II	17 (12 - 22)	21 (9.25 - 22.75)	19.5 (13.5 - 21.75)	0.6457
SOFA	7 (4.5 - 8.0)	8 (7.0 - 9.0)	8 (7.0 - 9.0)	0.1480
Shock	25 (71.4)	14 (77.8)	25 (83.3)	0.5213
Respiratory failure	26 (74.3)	14 (77.8)	26 (86.7)	0.4577
Vancomycin dosage (mg/dia)	2000 (2000 - 2000)	2000 (2000 - 2000)	2000 (2000 - 2000)	0.7178
Vancomycin dosage (mg/kg/dia)	32.1 (25.0 - 40.0)	29.3 (28.2 - 34.5)	27.2 (25.0 - 31.6)	0.3039

eGFR - estimated glomerular filtration rate; AKI - acute kidney injury; APACHE II - Acute Physiology and Chronic Health Disease Classification System II; SOFA - Sequential Organ Failure Assessment;

* Kruskal Wallis test followed to Dunn test for multiple comparison: significant difference between adequate and high VTL groups;

† Chi-squared test, significant difference between high versus adequate and inferior VTL groups. The results are expressed as median [25 - 75%] or number (%).

Among the 35 patients with an initial VTL lower than 15µg/mL, 15 (42.86% of this group of patients; 18.10% of all studied patients) presented with an initial VTL lower than 10µg/mL. Sex, age, the presence of shock and respiratory failure, SOFA and APACHE II scores, vanco dosage/day and vanco dosage/kg/day, serum creatinine and eGFR were not different between the groups stratified according to functions of VTLs. Estimated body weight was higher in patients with an initial VTL in the high group (p = 0.039). The proportion of patients with AKI diagnosed by KDIGO was significantly higher in patients with a high VTL (p = 0.0055, even with significance for trend, p = 0.0023), and, apparently, there seemed to be a greater number of cases in more advanced stages of AKI in patients with a high VTL.

Comparisons of clinical and laboratory characteristics of patients with and without a diagnosis of AKI are presented in table 2. Thirty-one patients (37.35% of sample) presented with AKI at the time of the first VTL determination. The daily vanco dosage was not different between patients with and without AKI, but patients with AKI presented with higher VTL levels (p = 0.0052). For all other tested parameters, no significant differences were found between the two groups.

**Table 2:** Comparison between basal clinical and laboratory data of patients with and without acute kidney injury

	AKI present (N = 31)	AKI absent (N = 52)	p value
VTL	21.60 (15.15 - 26.55)	13.85 (10.62 - 19.38)	0.0052*
Age	58 (47 - 72)	57 (40 - 68)	0.4978
Sex	20 (65)	34 (65)	0.9360
Weight (kg)	75 (70 - 80)	70 (65 - 80)	0.1022
Serum creatinine (mg/dL)	1.0 (0.9 - 1.3)	1.0 (0.8-1.2)	0.5203
eGFR (mL/min/1.73 m^2^)	74.22 (63.53 - 84.90)	79.55 (70.37 - 88.73)	0.4589
APACHE II	20 (11 - 22)	18 (13 - 22)	0.8541
SOFA	8 (6 - 9)	8 (6 - 9)	0.8305
Shock	27 (87)	37 (71)	0.1122
Respiratory failure	26 (84)	40 (77)	0.5776
Vancomycin dosage (mg/dia)	2000 (2000 - 2250)	2000 (2000 - 3000)	0.6567
Vancomycin dosage (mg/kg/ia)	28.6 (25.0 - 33.2)	30.4 (26.7 - 38.1)	0.1639

AKI - acute kidney injury; VTL - vancomycin trough levels; eGFR - estimated glomerular filtration rate; APACHE II - Acute Physiology and Chronic Health Disease Classification System II; SOFA - Sequential Organ Failure Assessment.

* Significant differences between patients with and without AKI by the Mann-Whitney test. The results are expressed as median [25 - 75%], number (%) or mean (CI95%).

Regarding VTL results, the initial VTL, as it was an inclusion criterion for the study, were available for the 83 studied patients. A second VTL and a third VTL were available for 58 and 26 patients, respectively, until the sixth day of follow up (losses were due to death, discharge from ICU or discontinuation of vanco). Due to the relatively small number of patients with 3 VTL determinations, statistical comparisons between VTL groups were only possible for the initial versus second VTL. The results from the Stuart-Maxwell's test showed that, compared to the initial VTL, the percentage of low VTL decreased from 27 (46.6%) to 14 (24.1%), and this decrease was due to an increase in high VTL (from 21 - 36.2% to 36 - 62.1%) and not to an increase in the proportion of adequate VTLs, which remained stable (changing from 10 - 17.2% - to 8 - 13.8%; p-value = 0.01582).

## DISCUSSION

In this study, analysis of initial VTLs showed that only 21.7% of the patients were within currently proposed targets; in 36.1% of the patients, they were elevated and, of note, they were below desired targets in 42.2% of patients. A subtherapeutic initial VTL in critically ill patients is a cause of concern, since delaying antibiotic therapeutic level achievement is associated with less favorable infectious outcomes and antimicrobial resistance.^(16)^

The finding of initial low VTLs in our study is not surprising, as a group of critically ill patients, especially young patients without many comorbidities, presented with augmented renal clearance, which is attributed to a hyperdynamic cardiovascular response (high cardiac output) secondary to a systemic inflammatory response. Such an increase in cardiac output leads to intensification in perfusion of different organs, including kidneys, and consequently in patients with adequate kidney reserve, GFR becomes raised. In this way, drugs excreted mainly by glomerular filtration, such as vanco, will have an increased rate of elimination from the body.^(17)^

Low VTLs could also be a consequence of severe obesity and, in septic patients, due to a capillary leak syndrome secondary to endotoxin liberation by microorganisms, which causes fluid shifting from an intravascular compartment to the interstitial space, increasing the volume of the distribution of the hydrophylic drug, as this is also the case with vanco.^(17)^ In our study, patients with an initial VTL in the high group had higher estimated body weights (p = 0.029). It should be stated that the median of patients' body weights were relatively low in the studied patients, and unmeasured variables (as for instance the low accuracy of the ICU team in estimating a patient´s weight^(18)^) could have accounted for this finding.

It should be noted that, as recommended for critically ill patients, a loading dose of 25 - 30mg of estimated weight was prescribed to our patients. Some authors suggested that continuous administration of a drug with a specific nomogram should be used to diminish the time to achieve adequate vanco levels in critically ill patients.^(19)^ Caution has been suggested when using continuous vanco infusions in obese patients, as lower maintenance doses seem to be necessary.^(20)^

As vanco nephrotoxicity must also be considered, a recently published systematic review and meta-analysis showed that adult patients treated with continuously administered vanco had a significantly lower incidence of nephrotoxicity compared with patients receiving the drug intermittently [risk ratio (RR) = 0.61, 95% confidence interval (CI) 0.47 - 0.80; p < 0.001], with no difference in treatment failures or patient mortality.^(21)^

There were no differences in age, sex, baseline serum creatinine, estimated GFR, APACHE II, SOFA, use of vasoactive drugs, need of mechanical ventilation, daily dosage of vanco and daily dosage of vanco per kg of weight between VTL groups. In the VTL groups, there was a difference between patients with a high VTL compared to the other two groups (p = 0.0055), and a tendency for a higher AKI proportion with a greater VTL category (p = 0.0023, chi-squared for trend). There is concern that a recommended VTL may be associated with a higher incidence of nephrotoxicity, especially when the VTL exceeds 20µg/mL or even 15µg/mL.^(22-25)^ There is, however, controversy if an elevated VTL observed in patients with AKI was the cause or consequence of AKI.^(26,27)^

Our results validated a criteria importance (as KDIGO) for a diagnosis of AKI,^(28)^ instead of using only the serum creatinine value. Indeed, serum creatinine concentrations or estimated GFR (eGFR) did not differ between the groups with and without AKI or between groups with a low, adequate or high VTL. Although commonly used in clinical practice, GFR estimation is not recommended in patients with unstable renal function,^(29)^ as patients with AKI, and it must be analyzed concomitantly with observation of diuresis (e.g., GFR in patients with 1 mg/dL creatinine, regardless of gender, age or weight, in an anuria state is zero).

In relation to age, this study found no significant difference between the median ages of patients with a low, adequate or high VTL. This result is in contrast with the findings of Legal and Wan^(30)^ who reported that younger patients exhibited a higher GFR, requiring lower vanco dose intervals (q8 h) to achieve therapeutic levels. A relatively small number of patients included in each of the three groups of VTL in the present study may have interfered with the analysis of this variable, as there was a trend to a smaller median age in patients with a low VTL - 53 (IQR 29 - 64) compared to adequate 64.5 (52.3 - 79.5) and high 55.5 (44.0 - 70.8) VTL values (p = 0.067).

AKI in ICU patients using vanco may be due to various causes other than nephrotoxicity, for example, severity of the disease, concomitant use of nephrotoxic agents and fluctuations in blood volume.^(31)^ Recently, the combined use of piperacillin/tazobactam with vanco has been shown to increase nephrotoxicity.^(32)^

We acknowledge that our paper has limitations. For instance, we did not include data on the use of other possible nephrotoxic agents by patients. We also did not study causative infectious agents, and the patient´s weight was estimated rather than measured. Nevertheless, our study showed that, with use of the therapeutic vanco schedule, a significant proportion of critically ill patients receiving vanco did not reach the initial VTL that is currently considered to be adequate to treat serious staphylococcal infection.

## CONCLUSION

The results of our study showed that monitoring vancomycin trough levels significantly help with decreasing the percentage of low vancomycin trough levels. However, in our patients, this decrease in the percentage of low vancomycin trough levels over time was obtained with an increasing rate of high vancomycin trough levels rather than with adequate vancomycin trough levels, suggesting that possibly more frequently or daily, determinations of vancomycin trough levels would be desirable for similar intensive care unit patients.

Studies of vancomycin dose regimens, the method of administration, i.e., intermittent versus continuous, and timing for first vancomycin trough levels in intensive care unit patients are required to circumvent the problem of a very high proportion of patients failing to reach the minimum recommended initial vancomycin trough levels for treatment of severe infections caused by gram-positive bacteria. Vancomycin trough levels monitoring is also very important to avoid the unnecessary risk of vancomycin nephrotoxicity. Our results might also suggest that, in health services where serum vancomycin determinations are not available, alternative antibiotics should be used for the treatment of serious gram-positive infections in intensive care unit patients.
